# Shenling Baizhu Powder Inhibits RV-SA11-Induced Inflammation and Rotavirus Enteritis via TLR4/MyD88/NF-κB Signaling Pathway

**DOI:** 10.3389/fphar.2021.642685

**Published:** 2021-04-09

**Authors:** Xiaoyan Wang, Qian Yang, Xiaofeng Zhou, Ting Chen, Liwen Dou, Furong Wang, Wei Wang

**Affiliations:** ^1^ College of Traditional Chinese Medicine, Shandong University of Traditional Chinese Medicine, Jinan, China; ^2^ Rizhao Hospital of Traditional Chinese Medicine, Rizhao, China; ^3^ Linyi Traditional Chinese Medicine Hospital-Endoscopic Centre, Linyi, China; ^4^ The First Clinical Medical College of Shandong University of Traditional Chinese Medicine, Jinan, China; ^5^ Department of Spleen and Stomach Diseases, Affiliated Hospital of Shandong University of Traditional Chinese Medicine, Jinan, China

**Keywords:** rotavirus enteritis, shenling baizhu powder, RV-SA11, TLR4, MyD88, NF-κB

## Abstract

Rotavirus enteritis (RVE) is a common acute intestinal infectious disease caused by rotavirus infection. It is an important cause of death in children younger than 5 years worldwide. Shenling baizhu powder (SBP), a classic traditional Chinese formulation, is one of the most popularly prescribed medicines for digestive diseases. Clinical studies have revealed the protective effects of SBP on RVE. However, the potential mechanism is still unclear. In this study, we aimed to evaluate the anti-rotavirus effect of SBP and its mechanism, focusing on the TLR4/MyD88/NF-κB signaling pathway. Our results demonstrated that, based on the inhibition of the virus-induced cytopathic effect in Caco-2 cells, the concentration for 50% of maximal effect (EC_50_) and selectivity index (SI) of SBP for RV-SA11 in the serum were 5.911% and 11.63, respectively. A total of 219 active compounds with oral bioavailability ≥30% and drug-likeness ≥ 0.18 were selected from the 10 ingredients present in the formulation of SBP, which acted on 471 potential targets. A total of 226 target genes of RVE were obtained from the GeneCards database. The protein-protein interaction (PPI) network showed that there was a close interaction between 44 common targets of SBP and RVE. The results of Gene Ontology (GO) and Kyoto Encyclopedia of Genes and Genomes (KEGG) enrichment analysis showed that SBP acted on RVE through various inflammatory pathways and the intestinal immune network. Subsequently, we investigated the effect of SBP on TLR4/MyD88/NF-κB signaling pathway *in vitro*. After infection with RV- SA11, the expression of TLR4, MyD88, and NF-κB mRNA and protein increased significantly, which could be abolished by SBP treatment. In addition, the IL-1β, TNF-α, IL-6, and IFN-β levels increased markedly in Caco-2 cells infected with RV-SV11. Treatment with SBP partly reversed the changes of IL-1β, TNF-α, and IL-6, while further increased the level of IFN-β. In conclusion, our study revealed that SBP can significantly inhibit rotavirus replication and proliferation *in vitro*. The antiviral effect may be related to the regulation of the TLR4/MyD88/NF-κB signaling pathway, followed by the down regulation of inflammatory cytokines and up regulation of IFN-β induced by rotavirus.

## Introduction

Rotavirus (RV), a double-stranded RNA virus belonging to the family Reoviridae, is the main cause of acute gastroenteritis in infants worldwide. In 2016, more than 258 million children younger than 5 years were infected with rotavirus with an annual incidence of 0.42 cases per child (Troeger et al., 2018). RV is also the leading infectious cause of diarrhea related deaths in children under 5 years old in the world; it accounts for 37% of the total number of diarrhea related deaths (Sestak, 2018). There were about 2,15,000 children who died of rotavirus enteritis in 2013; out of which more than 95% occurred in African and Asian countries (Tate et al., 2016). Up to date, neither WHO nor UNICEF has recommended effective drugs against RVE except for low osmolarity oral rehydration salts solution to prevent dehydration and zinc to reduce the duration and severity of diarrhea episodes. Since 2006, two live attenuated rotavirus vaccines (Rotarix^R^™, RotaTeq^R^™) have been licensed in more than 100 countries. However, the efficacy of rotavirus vaccine is only 50–60% in low-income countries and 80–90% in high-income countries (Burnett et al., 2018; Carvalho and Gill, 2018). Therefore, it is of great importance to develop effective and safe anti-rotavirus drugs.

Traditional Chinese medicine (TCM) offers unique advantages in maintaining health; thus, it has been widely used for thousands of years (Gu and Pei, 2017). Shenling baizhu powder is a classical formulation used in the treatment of gastrointestinal diseases, and has effects of supplementing Qi (energy), strengthening the spleen, and relieving diarrhea. SBP was first discovered in Taiping Huimin Heji Ju Fang compounded by Song Dynasty officials (1,078–1085 A.D.) and was recorded in the Pharmacopoeia of the People’s Republic of China, 2020. Full details of the main components of SBP, including family, vernacular name, TCM functions and so on were summarized in Table 1. Recent studies have reported that SBP has good efficacy and safety in treating rotavirus enteritis (Pu, 2017; Chen, 2018a; Qi, 2019). However, the anti-rotavirus effect and its mechanism of SBP have not been reported.

**TABLE 1:** Details of the SBP formula.

Species	Family	Vernacular name	Part used	TCM	Proportion	Batch code
Panax ginseng C.A.Mey.	Araliaceae Juss	Ren Shen	Root	1	10	1809021
Wolfiporia cocos (F.A. Wolf) Ryvarden and Gilb. (Poria cocos (Schw.) Wolf)	Laetiporaaceae Jülich	Fu Ling	Sclerotium	1	10	2010054
Atractylodes macrocephala Koidz.	Asteraceae Bercht and J.Presl	Bai Zhu	Rhizome	1	10	2008051
Dioscorea oppositifolia L.(Dioscorea opposita Thunb.)	Dioscoreaceae R.Br.	Shan yao	Tuber	2	10	2010007
Lablab purpureus (L.) Sweet (Dolichos lablab L.)	Fabaceae Linld.	Bai Bian Dou	Seed	2	7.5	2008079
Nelumbo nucifera Gaertn.	Nelumbonaceae A.Rich.	Lian Zi	Seed	2	5	2010077
Coix lacryma-jobi var. ma-yuen (Rom.Caill.) Stapf	Poaceae Barnhart	Yi Yi Ren	Fruit	2	5	1901090
Platycodon grandiflorus (Jacq.) A.DC.	Campanulaceae Juss.	Jie Geng	Root	3	5	1807003
Wurfbainia villosa (Lour.) Skornick. and A.D.Poulsen (Amomum villosum Lour.)	Zingiberaceae Martinov	Sha Ren	Fruit	4	5	1912096
Glycyrrhiza uralensis Fisch.	Fabaceae Lindl.	Gan Cao	Root	4	10	1812028

Table 1 Functions of the ingredients in the SBP formula, as attributed in the TCM: 1, are the main ingredients that have the effect of tonifying Qi, invigorating the spleen, and eliminating moisture. 2, can invigorate the spleen, relieve diarrhea, and enhance the efficacy of the active ingredient. 3, circulates Qi. 4, maintain the functions of the spleen and stomach. The nomenclature for plant species in this paper follows the accepted names by IPNI. (2021) and POWO. (2021) and for fungi those accepted by Justo et al. (2017) and Fungorum Species. (2021).

Network pharmacology is a systematic analysis of diseases, genes, protein targets, and drug interaction networks that reveal the synergistic effect of multiple molecular drugs (Hopkins et al., 2008; Kibble et al., 2015). Network pharmacology provides an alternative to explore the complex mechanisms of actions of traditional Chinese medicine. Therefore, in this study, we first predicted the active compounds, potential targets, and the signal pathways of SBP based on network pharmacology-based prediction. Additionally, we evaluated the anti-rotavirus effect and possible mechanisms of SBP in Caco-2 cells infected with simian rotavirus (SA-11) (Knipping et al., 2012).

## Materials and Methods

### Reagents and Antibodies

TRIzol and fetal bovine serum (FBS) were purchased from Shanghai ExCell Bio Co., Ltd., China. The Reverse Transcription Kit was obtained from Takara Biological Engineering Co., Ltd., China. Cell counting kit-8 (CCK-8) was obtained from Dojindo Laboratories, Kumamoto, Japan. Antibodies against TLR4, MyD88, and NF-κB p65, primary antibodies, and β-actin were obtained from Abcam (Cambridge, United Kingdom). ELISA kits for TNF-α, IL-1β, IL-6, and IFN-β were purchased from Boster Biological Technology Co., Ltd., China. Modified eagle’s medium (MEM) was obtained from Procell Life Science and Technology Co., Ltd., China. A 1% penicillin-streptomycin mixture and Electrochemiluminescence (ECL) reagent were purchased from Beijing Solarbio Science and Technology Co., Ltd., China.

### Preparation of the Drugs

As previously reported, the proportion of SBP was according to recommendations specified in the Pharmacopoeia of the People’s Republic of China (2020 Edition), as shown in Table 1. All the herbs were purchased from Beijing Tong Ren Tang Co., Ltd (Beijing, China). These herbs were soaked in water at a ratio of 1:10 for half an hour and heated for 1 h. The ingredients were extracted twice. These extracts were combined, filtered, and concentrated to 1 g/ml using a rotary evaporator. Ribavirin granules (Qianjin Xiangjiang Pharmaceutical Co., Ltd.) were prepared with distilled water to a concentration of 5 mg/ml.

### Preparation of SBP-Containing Serum

Thirty male Wistar rats (4 week-old, 100–120 g) were purchased from the Medical Animal Test Center of Shandong University (Jinan, China). After adaptation for three days, the rats were randomly divided into three groups (ten rats per group): normal group, ribavirin group (40.5 mg/kg/d, p. o.) and SBP group (8.37 g/kg/day, p. o.). The rats in the normal group were given the corresponding amount of saline. At the end of the third day, all the rats were anesthetized. Serum samples were collected from the abdominal aorta and centrifuged at 3,000 rpm in 4°C for 10 min. A portion of the supernatant was analyzed using ultra-high-performance liquid chromatography-quadrupole/electrostatic field orbital hydrazine high resolution mass spectrometry (UPLC-Q-Orbitrap HRMS/MS), and the other portions were used for experimentation.

### UPLC-Q-Orbitrap HRMS/MS Analysis

The analysis was performed using UPLC-Q-Orbitrap HRMS/MS (UltiMate 3000 RS) and Q-Exactive High Resolution Mass Spectrometer, which were purchased from Thermo Fisher Technology (China) Co., Ltd.). Chromatographic separation was carried out on an RP-C18 column (150 × 2.1 mm, 1.8 μm) at 35°C. The mobile phase consisted of 1): 0.1% aqueous solution of formic acid and 2): 0.1% formic acid in acetonitrile. The elution gradient was as follows: 0–1 min, 2% of B; 1–5 min, 20% of B; 5–10 min, 50% of B; 10–15 min, 80% of B; 15–25 min, 90% of B; 25–30 min 2% of B. The flow rate was 0.3 ml/min. The MS acquisition was performed using the positive and negative ion-switching modes of scanning. Mass spectrometry detection method: Full MS/dd-MS_2_; ion source: electrospray ionization source. The flow rates of sheath gas and auxiliary gas was 40 arb (arbitrary units) and 15 arb respectively, at 350°C. The capillary temperature was 300°C. Spray voltage was 3.8 kV for positive ionization and 3.8 kV for negative ionization. The primary scanning resolution was 70,000 FWHM, and the secondary scanning resolution was 17,500 FWHM. The scan range was 150–2000 m/z.

### Active Compounds and Predicted Targets of SBP

Using the traditional Chinese medicine system pharmacology (TCMSP) (Ru et al., 2014) (https://tcmspw.com/tcmsp.php) and Encyclopedia of Traditional Chinese Medicine (ETCM) (Xu et al., 2018) (http://www.tcmip.cn/ETCM/index.php/Home/Index/) databases, we collected the data on the chemical components contained in the ten traditional Chinese herbs in the formulation of SBP, and selected the compounds with oral bioavailability ≥30% and drug-likeness ≥ 0.18 as the active compounds of SBP. Based on the relevant pharmacological research results in the literature, we included some pharmacologically active compounds in the study, although the inclusion criteria mentioned above was not met. The targets of these compounds were obtained through the TCMSP and SwissTarget (Daina et al., 2019) (http://www.swisstargetprediction.ch/) databases, and the target proteins were converted into gene names using the UniProt database (https://sparql.uniprot.org/). Related genes of RVE were obtained from the Genecard database (https://www.genecards.org/), and the drug-compound-target network of SBP was constructed using Cytoscape 3.7.2.

Using Bioinformatics and Evolutionary Genomics (http://bioinformatics.psb.ugent.be/webtools/Venn/), we constructed a Venn diagram to screen the targets in the intersection of the active compounds of SBP and RVE The above intersection targets were imported into Search Tool for the Retrieval of Interacting Genes/Proteins database (Szklarczyk et al., 2019) (https://string-db.org/) to construct a PPI network. Using Metascape (http://metascape.org/gp/index.html), we conducted Gene Ontology enrichment analysis and Kyoto Encyclopedia of Genes and Genomes enrichment analysis of the targets in the intersection, and prepared a bar graph for the enrichment analysis. Based on the KEGG enrichment analysis and annotation results, the target-pathway network diagram was drawn.

### Cells and Virus

Caco-2 cells (Cell Bank of Type Culture Collection of the Chinese Academy of Sciences, Shanghai, China) were cultured in an MEM supplemented with 20% FBS and 1% penicillin-streptomycin mixture at 37°C and 5% CO_2_. The RV-SA11 strain (Institute of Viral disease Control and Prevention of the Chinese Center for disease Control and Prevention) was cultivated in Caco-2 cells in a medium containing 2% FBS and 1% penicillin-streptomycin mixture. The 50% tissue culture infective dose (TCID50) was calculated using the Reed–Muench method (TCID50 = 10–^4.31^/100 µL, Supplementary Table S1) (Reed and Muench, 1938).

### Cytotoxicity Test

The cytotoxicity of SBP-containing serum against Caco-2 cells was determined using CCK-8 assays (Zhang et al., 2018). Caco-2 cells (3 × 10^4^ cells/well) were seeded into a 96-well plate and incubated for 24 h. The cells were then treated with the drug-containing serum at serial concentrations of 50%, 40%, 30%, 20%, 10%, 5%, and 2.5%. The samples were incubated in 5% CO_2_ at 37°C for 24 h, then 10 μL CCK-8 was added into each well and incubated for an additional 2 h. The optical density was measured at 450 nm using a microplate reader, and the 50% cytotoxic concentration (CC50) of the medicated serum causing 50% of the cytopathic effect (CPE) of Caco-2 cells was calculated (Mosmann, 1983). All the experiments were performed in triplicate.

### Antiviral Effects of SBP-Containing Serum

Confluent monolayers of Caco-2 cells were inoculated with 100 TCID50/mL of RV-SA11 strain virus solution in a 96-well plate (100 μL per well) and adsorbed at 37°C for 2 h. The supernatant with the virus was discarded, and different concentrations (40%, 30%, 20%, 10%, 5%, and 2.5%) of SBP-containing serum (100 μL per well) were added. All of these were below the toxic concentration of the drug according to the cytotoxicity test results. Three wells were set for each concentration, normal cell control as well as virus control simultaneously. The CPE was observed daily, and the OD was detected using the CCK-8 method when CPE of the virus control was more than 90%. According to the results, the concentration of serum containing 20% of the drug was used in the follow-up experiment.

### Quantitative Real-Time Reverse Transcription PCR (RT-qPCR)

TRIzol was used to extract the total RNA from Caco-2 cells, and the cDNA was obtained using reverse transcription of mRNA using the Reverse Transcription Kit. The qRT-PCR method was performed as follows: pre-denaturation at 95°C for 10 min, denaturation at 95°C for 20 s, annealing at 58°C for 30 s, elongation at 72°C for 20 s, 40 cycles. The sequences of primers were as follows: β-actin: Forward 5′-CCC​ATC​TAT​GAG​GGT​TAC​GC-3′, Reverse 5′-TTT​AAT​GTC​ACG​CAC​GAT​TTC-3′; TLR4: Forward 5′-AGG​TTT​CCA​TAA​AAG​CCG​AAA​G-3′, Reverse 5′-CAA​TGA​AGA​TGA​TAC​CAG​CAC​G-3′; MyD88: Forward 5′-CGC​CGC​CTG​TCT​CTG​TTC​TTG-3′, Reverse 5′-GGT​CCG​CTT​GTG​TCT​CCA​GTT​G-3′; NF-κB p65: Forward 5′-ACA​GAA​GCA​GGC​TGG​AGG​TAA​GG-3′, Reverse 5′-GGA​CAA​TGC​CAG​TGC​CAT​ACA​GG-3′. The relative expression levels of mRNA were calculated by the 2^−△△CT^ method.

### Western Blot

Cells were lyzed to obtain protein samples, and the protein concentrations of the samples were detected using the BCA Protein Assay Kit (Beijing Solarbio Science and Technology Co., Ltd., China). Proteins from cell lysates were separated on 10% sodium dodecyl sulfate-polyacrylamide gel and then transferred onto a polyvinylidene fluoride membrane (Millipore, United States). The membranes were washed three times with Tris-buffered saline with 0.1% Tween^®^ 20 Detergent (TBST), blocked with 5% bovine serum albumin for 3 h at 25°C, and then incubated overnight with the corresponding primary antibodies at 4°C. The membranes were washed five times with TBST and incubated with secondary antibodies for 1 h at 25°C. Subsequently, after washing five times with TBST, we used an ECL reagent to capture the protein signals, and ImageJ software (NIH, United States) was used to visualize the protein bands. All experiments were performed in triplicate.

### Enzyme-Linked Immunosorbent Assay (ELISA)

The levels of inflammatory cytokines, including TNF-α, IL-1β, IL-6, and IFN-β in the supernatant were determined using ELISA kits. The experimental steps were performed according to the manufacturer’s instructions. The OD was measured using a microplate reader at 450 nm.

### Statistical Analysis

All data in the experiments were tested for normality, and were conformed to normal distribution. The data were expressed as mean ± standard deviation. SPSS 19.0 software (IBM, Chicago, IL, United States) was used for one-way ANOVA followed by the Fisher’s least significant difference (LSD) test for multiple comparisons. A *p* value <0.05 was considered to be a statistically significant difference between the groups.

## Results

### Chemical Constituents of SBP-Containing Serum

**FIGURE 1:**
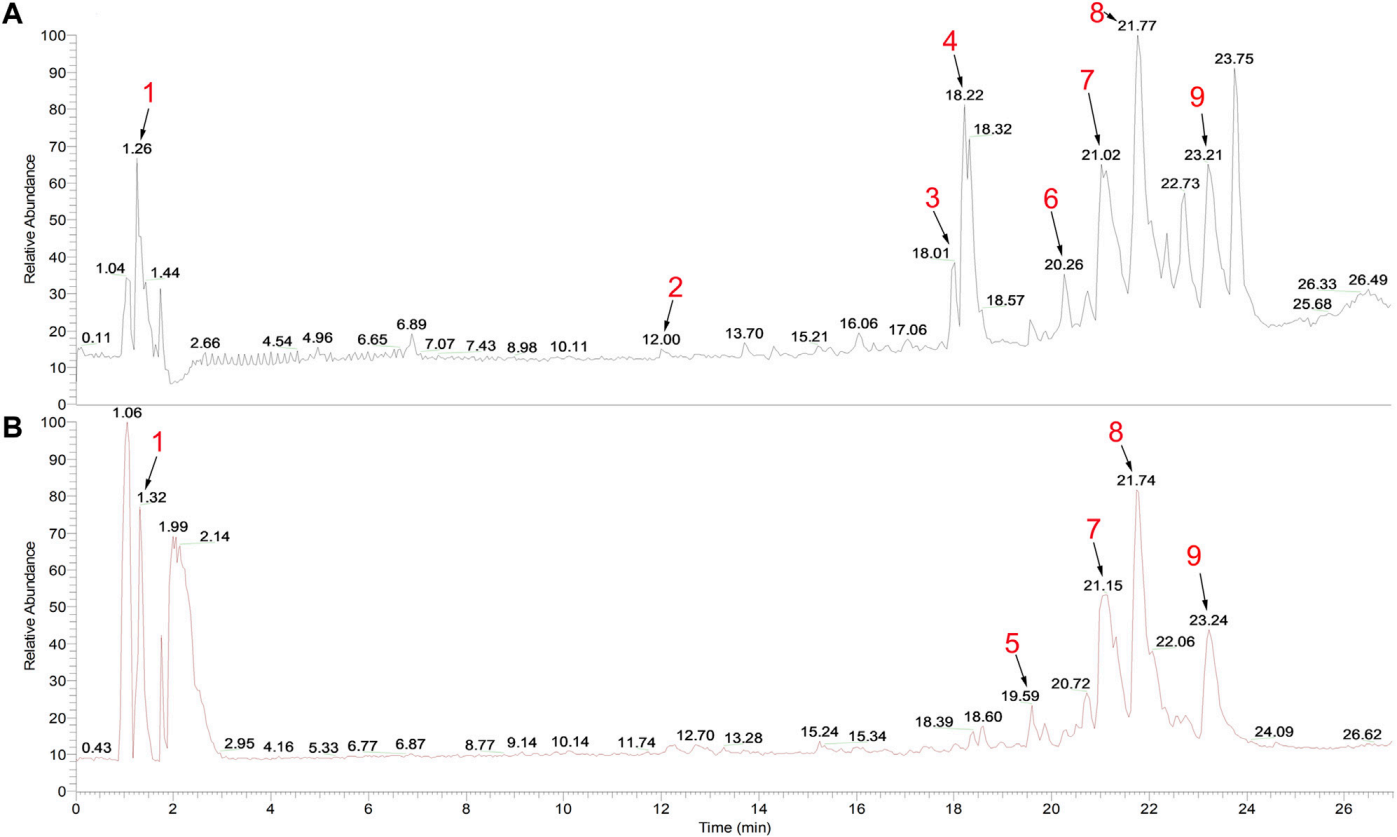
Total ion chromatogram of SBP-containing serum in **(A)** positive and **(B)** negative modes. Peak 1: N1-(1,3,5-Trimethyl-1H-pyrazol-4-yl)-2-cyano-3-(dimethylamino) acrylamide; 2: isoliquiritigenin; 3: 2-Methoxyestrone; 4: 6-O-Pentopyranosyl-1-O-[(2,6,6-trimethyl-1-cyclohexen-1-yl)carbonyl]-β-d-glucopyranose; 5: oleanolic acid; 6: monoolein; 7: 18-β-Glycyrrhetinic acid; 8: 9(Z),11**(E)**-Conjugated linoleic acid; and 9: oleic acid.

The chemical constituents of SBP-containing serum were analyzed using UPLC-Q-Orbitrap HRMS. The results are shown in Figure 1; Supplementary Table S2. A total of nine ingredients were identified using excimer peaks, information on fragmentation, and literature. These included, N1-(1,3,5-Trimethyl-1H-pyrazol-4-yl)-2-cyano-3-(dimethylamino) acrylamide, isoliquiritigenin, two Methoxyestrone, 6-O-Pentopyranosyl-1-O-[(2,6,6-trimethyl-1-cyclohexen-1-yl)carbonyl]-β-d-glucopyranose, oleonolic acid, monoolein, 18-β-Glycyrrhetinic acid, 9(Z),11(E)-Conjugated linoleic acid, and oleic acid.

### Active Compounds and Predicted Targets of SBP

**FIGURE 2:**
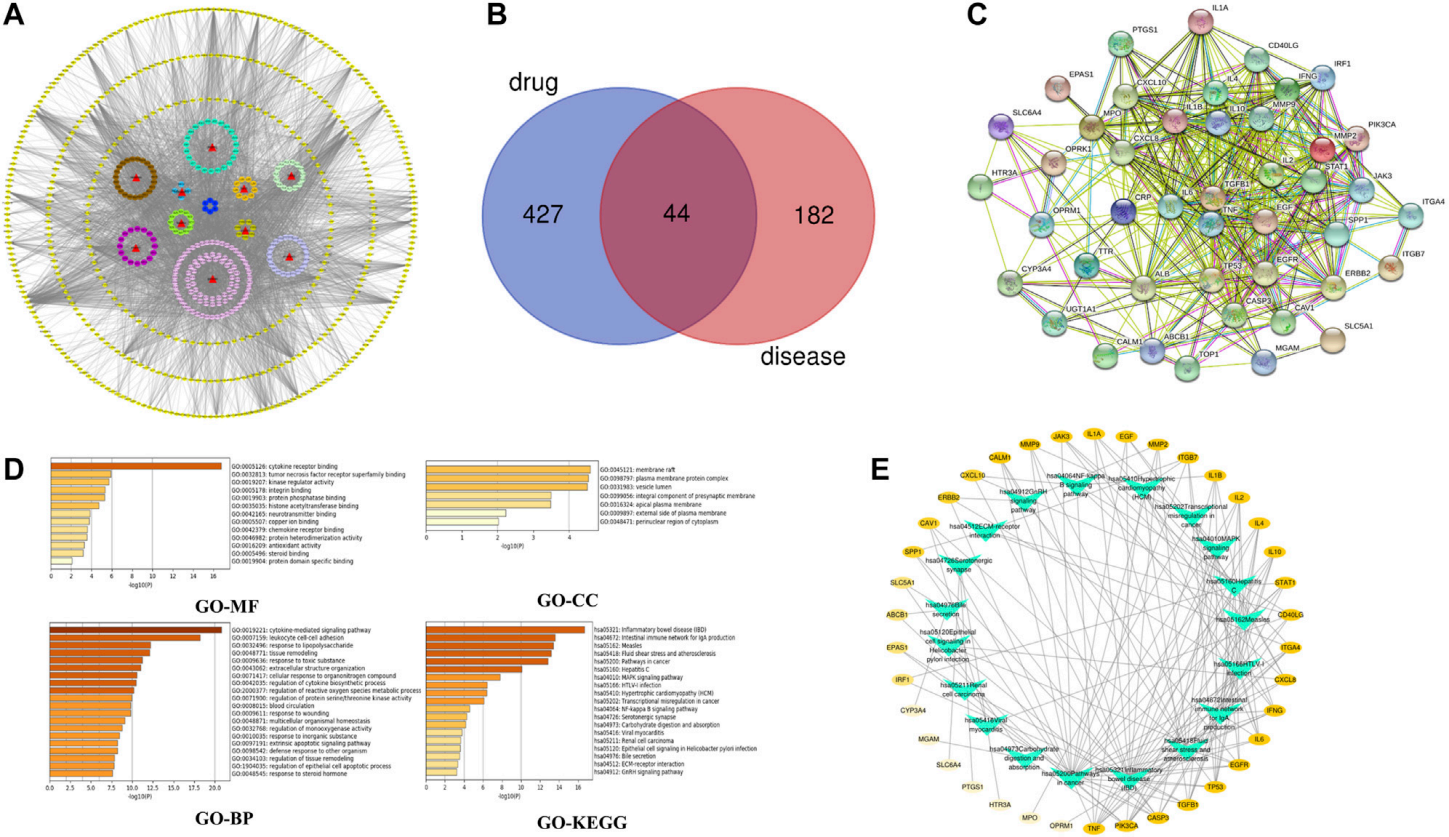
Network pharmacology analysis **(A)** Drug-compound-target network of the anti-rotavirus effect of SBP. 10 herbs are shown as red triangles, 219 active compounds are shown as circles with different colors, and 471 target genes are shown as yellow diamonds **(B)** The common targets of SBP and RVE **(C)** The PPI network of SBP-RVE **(D)** GO analysis and KEGG pathway analysis of the common targets of SBP and rotavirus using a bar graph to rank the importance from top to bottom **(E)** Target-Pathways network. The target genes of SBP-RVE are shown as a yellow oval node. The higher the degree of the target gene, the darker the color. Paths are marked with green triangle nodes.

According to the oral bioavailability ≥30% and drug-likeness ≥ 0.18, a total of 219 active compounds were selected from the 10 ingredients in the SBP formulation, and they acted on 471 potential targets. The drug-compound-target network constructed using Cytoscape 3.7.2 composed of 708 nodes and 3,921 edges (Figure 2A). From this network, we identified that several compounds act on multiple targets, and vice versa. A total of 226 target genes of RVE were obtained from the GeneCards database.

The Venn diagram showed that SBP and RVE have 44 common target proteins (Figure 2B), which might be the biological basis of SBP’s effect on RVE. The PPI network showed that there is a close interaction between these 44 common targets. This showed that RGFR, EGF, IL-6, TGFB1, TNF, IL-2, and INF were the key targets of this PPI network (Figure 2C).

GO and KEGG analyses were performed using the Metascape database (Figure 2D). GO enrichment analysis showed that SBP primarily acted on membrane rafts, plasma membrane-protein complexes and lumens of vesicle, and affected cytokine-mediated signaling pathways, cell-cell adhesion of leukocytes, and other biological processes by affecting the functions of cytokine-receptor binding. To show the relationship between 44 targets and their corresponding KEGG pathways, we constructed a target-pathway network (Figure 2E), and the results showed that SBP acted on RVE by influencing several inflammatory pathways and intestinal immune networks.

### Inhibitory Effects of SBP-Containing Serum on RV-SA11

**FIGURE 3:**
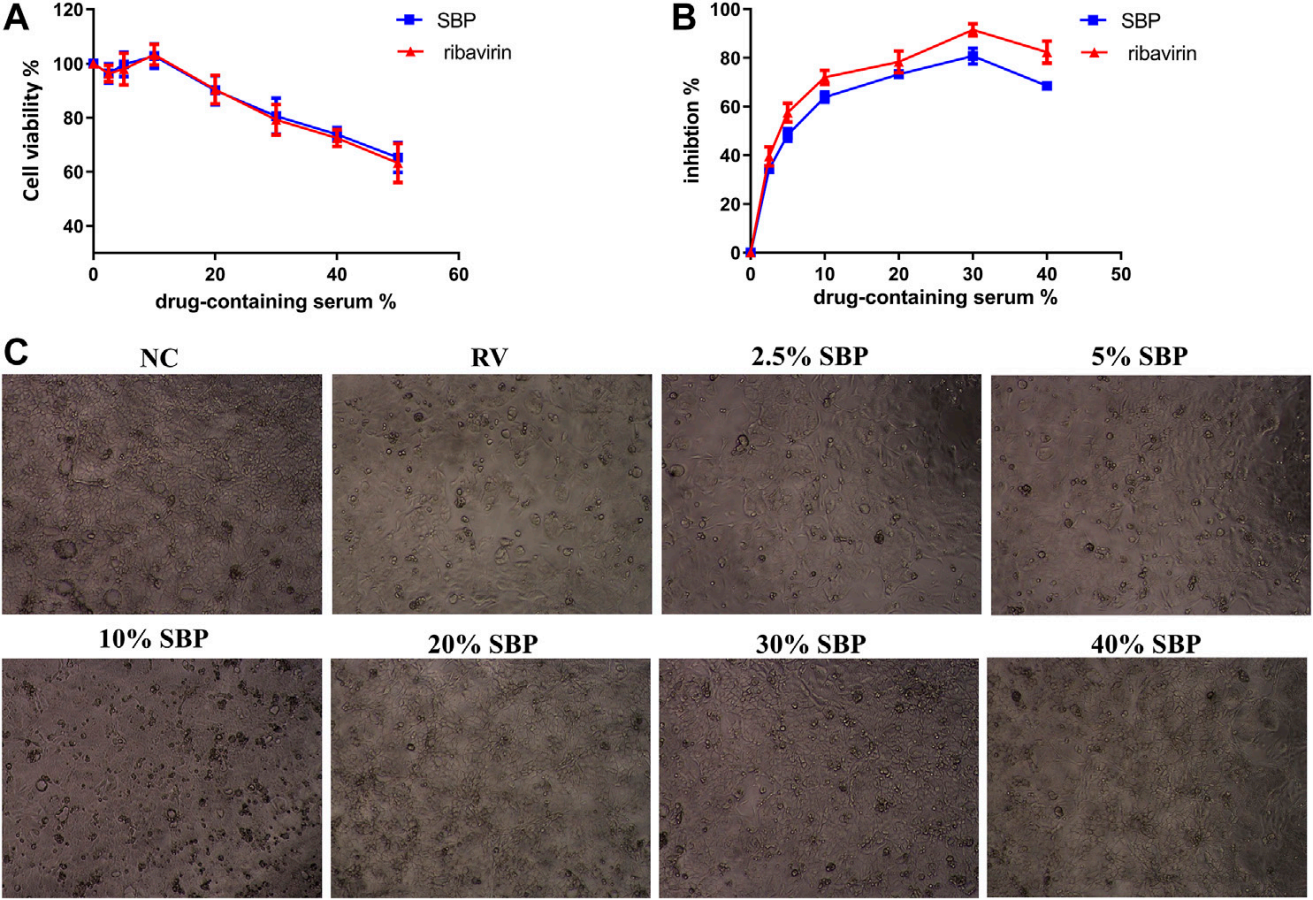
Inhibition of RV-SA11 activity in different concentrations of SBP-containing serum *in vitro* **(A)** The cytotoxic effects of the serum on Caco-2 cells was detected using a CCK-8 assay (*n* = 3) **(B)** The inhibitory effects of the serum on RV- SA11-infected Caco-2 cells. Error bars indicate the range of values obtained from the triplicate experiments; represented as mean ± SD of three individual experiments **(C)** The effect of the serum on the pathological changes in virus-infected Caco-2 cells (40 X).

To assess the antiviral efficacy of SBP, the inhibitory effects of SBP-containing serum on the replication and proliferation of RV-SA11 were tested. The CC50 of SBP-containing serum in Caco-2 cells was 68.75% (Figure 3A; Supplementary Table S3). The EC50 value of SBP-containing serum was 5.911% (Figure 3B; Supplementary Table S4)), and the SI was 11.63. The CC50 of ribavirin-containing serum in Caco-2 cells was 65.08% (Figure 3A). The EC50 value of ribavirin-containing serum was 3.467% (Figure 3B) and the SI was 18.77. The results showed that both SBP and ribavirin significantly inhibited virus replication and proliferation with less toxic effects on the viability of Caco-2 cells. Meanwhile, we examined the effects of SBP on the pathological changes in virus-infected Caco-2 cells (Figure 3C). The Caco-2 cells in the normal control group (NC) had uniform morphology with irregular and oval tight junctions and clear cell margins. The cells of the RV group were uneven in shape with vacuoles, large intercellular space, blurred margins, and dead cells and cell debris. The drug-containing serum acts on rotavirus-infected cells. As we increased the concentration of the drug-containing serum, the number of cell vacuoles and dead cells decreased, the cell margins became clearer, and the connections were tighter.

### Effect of SBP on TLR4/MyD88/NF-κB Signaling Pathway

**FIGURE 4:**
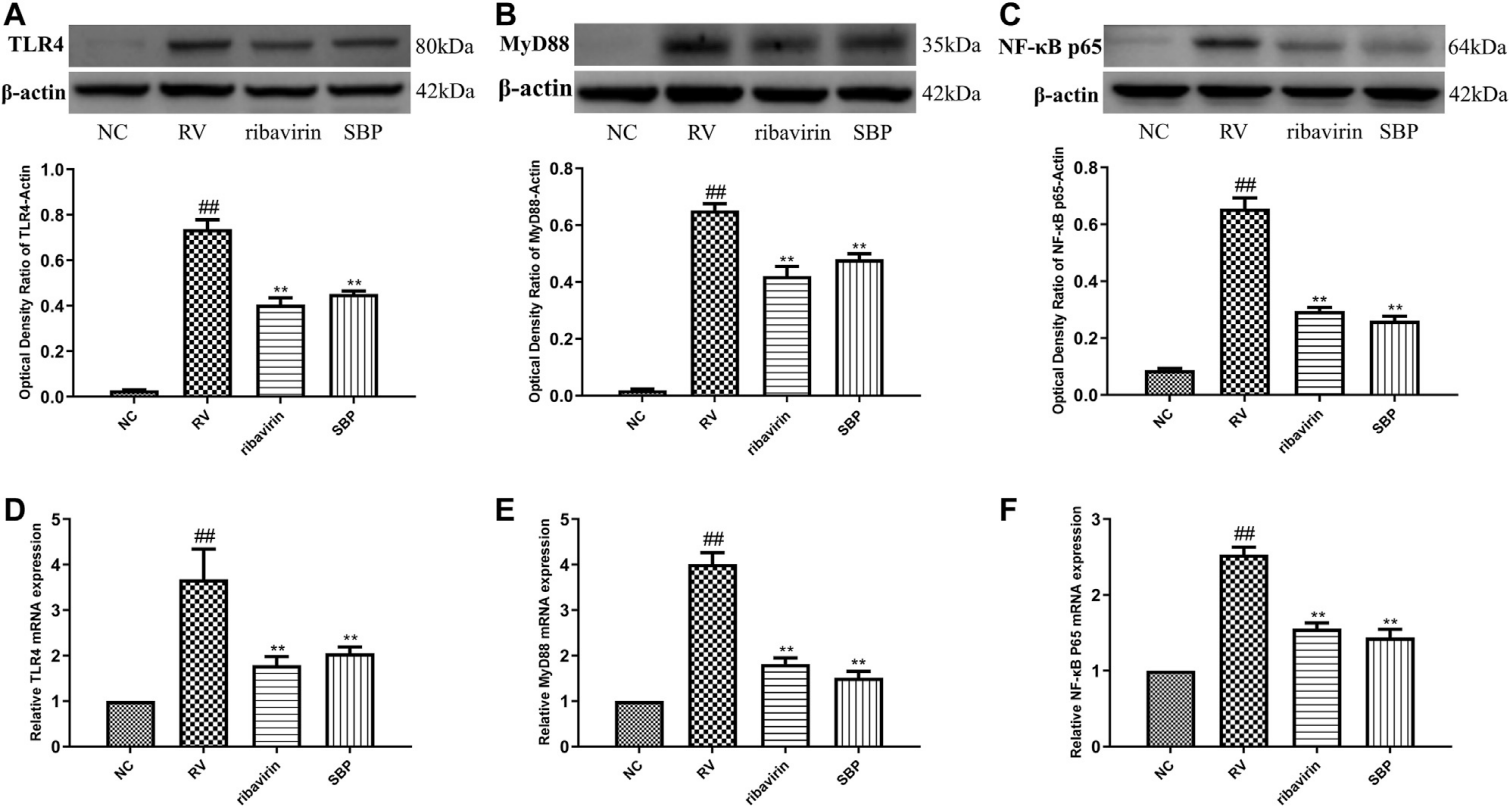
The mRNA and protein expressions of TLR4, MyD88, and NF-κB p65 **(A–C)** Western blot was carried out to measure the expressions of TLR4, MyD88, and NF-κB p65, and ImageJ 1.8.0 112 software was used to analyze and transform the statistical data of all the proteins (n = 3) **(D–F)** RT-qPCR for mRNA expression of TLR4, MyD88, and NF-κB p65 (*n* = 3). All values were presented as mean ± SD. ^##^ *p* < 0.01 vs. NC group; *P <0.05, **p < 0.01 vs. RV group.

To investigate the mechanism of SBP on RV, we detected the mRNA and protein expressions of TLR4 and downstream MyD88 and NF-κB p65. The mRNA and protein expressions of TLR4, MyD88, and NF-κB p65 were significantly increased in RV-SA11-infected Caco-2 cells (*p* < 0.01). SBP significantly decreased the mRNA and protein expressions of TLR4, MyD88, and NF-κB p65 induced by RV-SA11 (*p* < 0.01). This indicates that SBP inhibits RV-SA11 virus by inhibiting the TLR4/MyD88/NF-κB signaling pathway (Figure 4; Supplementary Tables S5, 6).

### Effect of SBP-Containing Serum on Inflammation of the Virus Infected Cells

**FIGURE 5:**
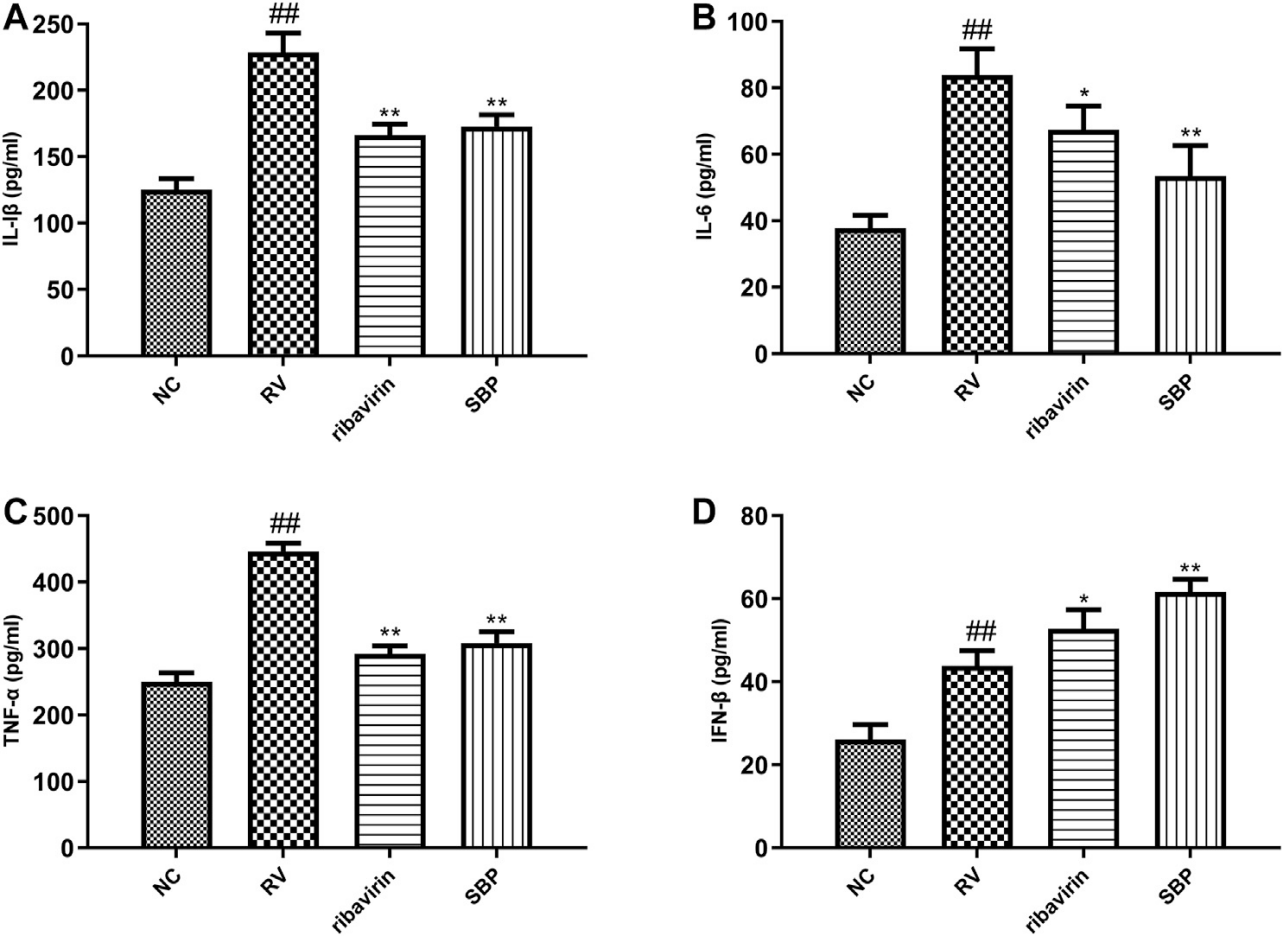
The expression of cytokines detected using ELISA (*n* = 3). All values were presented as mean ± SD. ^##^ *p* < 0.01 vs. NC group; *p < 0.05, **p < 0.01 vs. RV group.

To further study the effect of SBP on the inflammatory response induced by RV-SA11, ELISA was used to detect the production of cytokines in the supernatant of the infected cells. The expression levels of IL-1β, IL-6, TNF-α, and IFN-β in the RV group were significantly higher than those in the NC group (*p* < 0.01). The expression levels of IL-1β, IL-6, and TNF-α in the SBP group were significantly decreased (*p* < 0.01) when compared to the RV group (Figures 5A–C; Supplementary Table S7). Meanwhile, the expression level of IFN-β further increased in the SBP group to play its antiviral role (Figure 5D; Supplementary Table S7). These results showed that SBP was able to regulate the overexpression of various inflammatory cytokines in the RV-SA11-infected Caco-2 cells.

## Discussion

Caco-2 cells are cloned human colon adenocarcinoma cells that are structurally and functionally similar to differentiated small intestinal epithelial cells, and are commonly used cell lines for research of RV infection (Knipping et al., 2012). In this study, we found that SBP-containing serum had less toxic effects on Caco-2 cells and a strong inhibitory effect on RV-SA11, indicating that SBP has a good anti-rotavirus effect. Our data are in line with previous studies showing that the multiple active ingredients in SBP have antiviral properties. Ginsenoside-Rb2 and its hydrolysate 20(S)-ginsenoside Rg3 inhibited the growth of RV and had a protective effect on infected cells (Yang et al., 2018a). Glycyrrhizic acid and its primary metabolite 18β-glycyrrhetinic acid (GRA) are pharmacologically active compounds of Glycyrrhiza uralensis Fisch, and both have antiviral and immunomodulatory properties. GRA had significant antiviral activity against rotavirus replication *in vitro* and reduced the levels of viral proteins VP2, VP6, and NSP2 in the infected cells (Hardy, et al., 2012). Atractylodes lactone III exerted antiviral effects by directly inactivating RV *in vivo* and *in vitro* (Zhou et al., 2019). The triterpenoid saponins from Platycodon grandiflorus (Jacq.) A.DC. suppressed activity against HCV replication (Kim et al., 2013).

SBP is composed of 10 different Chinese herbs, and its active compounds are complex. It is difficult to clarify each of its mechanism using classical pharmacological techniques. Network pharmacology is a powerful tool to study the mechanism of such compounds in Chinese herbal formulations, and helps clarify complex biological phenomena. In order to determine the molecular mechanism of SBP, we constructed a drug-compound-target network, selected 44 drug-disease targets, and performed GO enrichment analysis and KEGG pathway analysis. The results suggested that SBP acted on RVE by influencing several inflammatory pathways and intestinal immune networks.

Numerous studies showed that RV infection promoted the production of inflammatory cytokines, such as IL-1β, IL-6, IL-8, and TNF-α (Ye et al., 2017; Hakim et al., 2018; Shen et al., 2019). The results of this study revealed that RV could up-regulate the expression of IL-1 β, TNF-α, and IL-6, which could be reversed using SBP. Ginsenoside Rg3 had an immunomodulatory effect, with studies showing that it could increase the production of anti-inflammatory cytokines (IL-2, IL-10, etc.), reduce the levels of pro-inflammatory mediators (TNF-α and IL-1β), and enhance immune function (Yang et al., 2018b; Liu et al., 2019). Isoliquiritigenin (ILG), a natural flavonoid, could inhibit inflammation-induced small intestinal damage by reducing the levels of cleaved caspase-1 and production of mature IL-1β (Nakamura et al., 2018). Multiple studies have shown that the compounds isolated from *Atractylodes macrocephala* Koidz., the glycoprotein of *Dioscorea opposita* Thunb., and platycodin D could reduce the levels of pro-inflammatory cytokines (IL-1β, IL-6 and TNF-α) via inhibition of NF-κB signaling pathways (Jeong et al., 2019; Niu et al., 2020; Ye et al., 2019).

Type I IFNs, such as IFN-α, IFN-β, and IFN-κ cytokines, are involved in the activation and regulation of innate and acquired immune responses. They have strong antiviral and immunomodulatory activities (Klotz et al., 2017). After the virus infects the cells, pattern recognition receptors (PRRs) activate the innate immune response. PRRs use specific adaptor proteins including MyD88 and Toll/IL-1R domain-containing adaptor-inducing IFN-β (TRIF) to activate interferon regulatory factor 3 (IRF3), IRF7, and NF-κB. These factors initiated the transcription of type I IFNs and pro-inflammatory cytokines (such as IL-1β, IL-6, and TNF-α) (Carty et al., 2014; Klotz et al., 2017). However, previous studies have shown that endogenous IFN cannot effectively limit the replication of RV. RV might inhibit the immune response of IFN through different mechanisms: The nonstructural protein of RV (NSP1) could inhibit the production of type 1 IFN by degrading IFN induced IRF transcription factors or inhibiting NF-κB activation; RV could isolate NF-κB in the virion and inhibit STAT nuclear accumulation stimulated by IFN (Sherry, 2009). Ultimately, the inhibition of IFN immune response depended on the RV strain and cell type (Sen et al., 2018). In our experiment, the expression of IFN-β increased in RV infected cells. After SBP treatment, the expression of IFN-β further increased, which played an antiviral role to a certain extent. Similarly, ginsenoside Rg3 enhanced innate immune response by inducing IFN-β expression through stimulating DEAD-box RNA helicase DDX3 expression via p53-mediated transactivation and activation of the TBK1/IKKɛ/IRF3 pathway (Choi et al., 2014). *Glycyrrhiza uralensis Fisch.* dose-dependently stimulated the secretion of IFN-β in A549 and Hep-2 cells with and without HRSV infection (Feng Yeh et al., 2013).

NF-κB is an important transcription activator that regulates the expression of inflammatory mediators. It was closely related to various types of inflammation and plays an important role in mediating immune responses (Mitchell et al., 2016). The activation of NF-κB could induce the production of cytokines, which could interact to aggravate inflammatory reactions (Zhang et al., 2020). We found that the expression of NF-κB p65 mRNA and protein was significantly increased in RV-SA11-infected Caco-2 cells, which significantly decreased after SBP treatment.

Toll-like receptors (TLRs) are a type of PRR that play an important role in the host immune system. They could recognize bacteria, viruses, and other microbial molecules and activate the body to produce immune cell responses (Chen et al., 2018b). Toll-like receptor 4 (TLR4), a member of the Toll-like family, is located in the cell membrane and cytoplasm, and is a PRR for lipopolysaccharides (LPS). The interaction between LPS and TLR4 promoted the activation of the downstream NF-κB signaling pathway, which eventually leaded to inflammation (Ciesielska et al., 2020). RV is a virus that can infect both humans and animals. It localizes in intestinal epithelial cells and induces an immune response through certain signaling pathways. Intestinal epithelial cells were important sites for the expression and coding of TLRs, IL-1 β, IL-6, and GM-CSF (Soderholm and Pedicord, 2019). RV could induce the activation of dendritic cells, which could upregulate the levels of CD40, CD86, TLR3, and TLR4, and produce inflammatory cytokines, such as IL-6, IL-10, TNF-α, and interferon-β (Rosales-Martinez et al., 2016; Ye et al., 2017). MyD88 is a key signal transduction protein involved in the TLR signaling pathway. Except for TLR3, all other TLRs use MyD88-dependent pathways. MyD88 could activate the NF-κB and induced the initiation of immune response after RV infection, including the release of pro-inflammatory cytokines and the expression of defense proteins (Saikh et al., 2020). It was found that the immune response of RV-infected mice lacking MyD88 was lower, which contributed to the infection and transmission of RV, and confirmed that MyD88-mediated TLR signaling pathway limited the infection and transmission of RV (Uchiyama et al., 2014). Therefore, regulation of the TLR4/MyD88/NF-κB signaling pathway may help reduce the inflammatory response. Here, we found that SBP obviously inhibited the mRNA and protein levels of TLR4 and MyD88 in RV-infected Caco-2 cells. ILG exerted antioxidative and anti-inflammatory effects via activating the KEAP-1/Nrf2 pathway and inhibiting the NF-κB and NLRP3 pathway (Gao et al., 2020). Oleanolic acid could alleviate diarrhea caused by *Salmonella typhimurium*, maintain the integrity of intestinal barrier via the TLR4/NF-κB and MAPK signaling pathway, and inhibit the secretion of pro-inflammatory cytokines such as TNF-α, IL-1β, and IL-6 (Dong et al., 2020). The fatty acids, esters, lactams and polyphenols in *Coix lacryma-jobi* var. *ma-yuen (Rom.Caill.) Stapf* had anti-inflammatory effects. The mechanism may be related to the decrease of vascular permeability and inflammatory exudation, the intervention of IKK/NF-κB signaling pathway, and the decrease of the secretion of inflammatory factors such as IL-6, CCl2, IL-1α, IL-1β (Li et al., 2020).

## Conclusion

In conclusion, our results indicated that SBP could significantly inhibit virus replication and proliferation with low cytotoxicity *in vitro*. The antiviral effect of SBP may be related to the regulation of the TLR4/MyD88/NF-κB signaling pathway, and prohibition of the release of cytokines caused by RV. These preliminary data could not fully explain the underlying mechanism of anti-rotavirus effect of SBP. Also the protective effects of SBP on RVE *in vivo* are absent. The present study has thrown new light on the drug therapy of RVE. Moreover, due to the complexity of components inTCM, new technologies are needed to investigate the material basis and detailed mechanism of SBP.

## Data Availability

The raw data supporting the conclusions of this will be made available by the authors, without undue reservation, to any qualified researcher.

## Abbreviation

CC50, 50% cytotoxic concentration; CPE, cytopathic effect; DL, drug-likeness; ECL, electrochemiluminescence; ELISA, Enzyme-linked immunosorbent assay; ETCM, Encyclopedia of Traditional Chinese Medicine; FBS, fetal bovine serum; GO, Gene Ontology; KEGG, Kyoto Encyclopedia of Genes and Genomes; LPS, lipopolysaccharides; LSD, least significant difference; MEM, Modified eagle’s medium; OD, optical density; RT-qPCR: Quantitative real-time reverse transcription PCR; RVE; Rotavirus enteritis, SBP; shenling baizhu powder, SI; selectivity index; TCMSP, traditional Chinese medicine system pharmacology; TLRs, toll-like receptors; UPLC-Q-Orbitrap HRMS/MS, ultra-high-performance liquid chromatography-quadrupole/electrostatic field orbital hydrazine high resolution mass spectrometry

## References

[B1] Burnett E. Parashar U. Tate J. (2018). Rotavirus vaccines: effectiveness, safety, and future directions. Pediatr. Drugs 20 (3), 223–233. 10.1007/s40272-018-0283-3 PMC5955791 29388076

[B2] Carty M. Reinert L. Paludan S. R. Bowie A. G. (2014). Innate antiviral signalling in the central nervous system. Trends Immunol. 35 (2), 79–87. 10.1016/j.it.2013.10.012 24316012

[B3] Carvalho M. F. Gill D. (2018). Rotavirus vaccine efficacy: current status and areas for improvement. Hum. Vaccin. Immunother. 15 (6), 1237–1250. 10.1080/21645515.2018.1520583 30215578 PMC6663136

[B4] Chen C.-Y. Kao C.-L. Liu C.-M. (2018b). The cancer prevention, anti-inflammatory and anti-oxidation of bioactive phytochemicals targeting the TLR4 signaling pathway. Ijms 19 (9), 2729–2745. 10.3390/ijms19092729 PMC6164406 30213077

[B5] Chen L. (2018a). Clinical effect of Shenling Baizhu Powder in the treatment of infantile autumn diarrhea. Clin. Med. Res. Pract. 3 (13), 124–125. 10.19347/j.cnki.2096-1413.201813059

[B6] Choi Y.-J. Kang L.-J. Lee S.-G. (2014). Stimulation of DDX3 expression by ginsenoside Rg3 through the akt/p53 pathway activates the innate immune response via TBK1/ikkε/IRF3 signalling. Cmc 21 (8), 1050–1060. 10.2174/09298673113206660306 24180280

[B7] Ciesielska A. Matyjek M. Kwiatkowska K. (2020). TLR4 and CD14 trafficking and its influence on LPS-induced pro-inflammatory signaling. Cell. Mol. Life Sci. 78, 1233–1261 10.1007/s00018-020-03656-y 33057840 PMC7904555

[B8] Daina A. Michielin O. Zoete V. (2019). SwissTargetPrediction: updated data and new features for efficient prediction of protein targets of small molecules. Nucleic Acids Res. 47 (W1), W357–W364. 10.1093/nar/gkz382 31106366 PMC6602486

[B9] Dong N. Xue C. Zhang L. Zhang T. Wang C. Bi C. (2020). Oleanolic acid enhances tight junctions and ameliorates inflammation in *Salmonella* typhimurium-induced diarrhea in mice via the TLR4/NF-κB and MAPK pathway. Food Funct. 11 (1), 1122–1132. 10.1039/c9fo01718f 31825448

[B10] Feng Yeh C. Chih Wang K. Chai Chiang L. Shieh D. E. Hong Yen M. San Chang J. (2013). Water extract of licorice had anti-viral activity against human respiratory syncytial virus in human respiratory tract cell lines. J. Ethnopharmacology 148 (2), 466–473. 10.1016/j.jep.2013.04.040 PMC7126896 23643542

[B11] Fungorum Species (2021). Search species Fungorum Availale at: https://www.speciesfungorum.org/Names/Names.asp

[B12] Gao Y. Lv X. Yang H. Peng L. Ci X. (2020). Isoliquiritigenin exerts antioxidative and anti-inflammatory effects via activating the KEAP-1/Nrf2 pathway and inhibiting the NF-κB and NLRP3 pathways in carrageenan-induced pleurisy. Food Funct. 11 (3), 2522–2534. 10.1039/c9fo01984g 32141447

[B13] Gu S. Pei J. (2017). Innovating Chinese herbal medicine: from traditional health practice to scientific drug discovery. Front. Pharmacol. 8, 381–385. 10.3389/fphar.2017.00381 28670279 PMC5472722

[B14] Hakim M. S. Chen S. Ding S. Yin Y. Ikram A. Ma X.-x. (2018). Basal interferon signaling and therapeutic use of interferons in controlling rotavirus infection in human intestinal cells and organoids. Sci. Rep. 8 (1), 8341–8353. 10.1038/s41598-018-26784-9 29844362 PMC5974418

[B15] Hardy M. E. Hendricks J. M. Paulson J. M. Faunce N. R. (2012). 18β-glycyrrhetinic acid inhibits rotavirus replication in culture. Virol. J. 9, 96–111. 10.1186/1743-422x-9-96 22616823 PMC3478227

[B16] Hopkins A. L. D. Dong G. Z. Lee H. J. Ryu J. H. (2008). Network pharmacology: the next paradigm in drug discoveryAnti-Inflammatory Compounds from Atractylodes macrocephala. Nat. Chem. Biolmolecules 4 (11), 682–690. 10.1038/nchembio.118

[B17] IPNI (2021). International plant name index (IPNI) Availale at: https://www.ipni.org.

[B52] Jeong D. Dong G. Z. Lee H. J. Ryu J. H. (2019). Anti-inflammatory compounds from Atractylodes macrocephala. Molecules 24 (10), 1859. 10.3390/molecules24101859 PMC6571718 31091823

[B18] Justo A. Miettinen O. Floudas D. Ortiz-Santana B. Sjökvist E. Lindner D. (2017). A revised family-level classification of the Polyporales (Basidiomycota). Fungal Biol. 121 (9), 798–824. 10.1016/j.funbio.2017.05.010 28800851

[B19] Kibble M. Saarinen N. Tang J. Wennerberg K. Mäkelä S. Aittokallio T. (2015). Network pharmacology applications to map the unexplored target space and therapeutic potential of natural products. Nat. Prod. Rep. 32 (8), 1249–1266. 10.1039/c5np00005j 26030402

[B20] Kim J.-W. Park S. J. Lim J. H. Yang J. W. Shin J. C. Lee S. W. (2013). Triterpenoid saponins isolated fromPlatycodon grandiflorumInhibit hepatitis C virus replication. Evidence-Based Complement. Altern. Med. 2013, 1–11. 10.1155/2013/560417 PMC3893781 24489585

[B21] Klotz D. Baumgärtner W. Gerhauser I. (2017). Type I interferons in the pathogenesis and treatment of canine diseases. Vet. Immunol. Immunopathology 191, 80–93. 10.1016/j.vetimm.2017.08.006 28895871

[B22] Knipping K. Garssen J. van’t Land B. (2012). An evaluation of the inhibitory effects against rotavirus infection of edible plant extracts. Virol. J. 9, 137–144. 10.1186/1743-422X-9-137 22834653 PMC3439294

[B23] Li X. Gu K. Liang M. Zhang Y. Wang Y. Li Y. (2020). Research progress on chemical constituents and pharmacological effects of Coicis Semen. Chin. Traditional Herbal Drugs 51 (21), 5645–5657. 10.7501/j.issn.0253-2670.2020.21.031

[B24] Liu X. Zhang Z. Liu J. Wang Y. Zhou Q. Wang S. (2019). Ginsenoside Rg3 improves cyclophosphamide-induced immunocompetence in Balb/c mice. Int. Immunopharmacology 72, 98–111. 10.1016/j.intimp.2019.04.003 30974284

[B25] Mitchell S. Vargas J. Hoffmann A. (2016). Signaling via the NFκB system. Wires Syst. Biol. Med. 8 (3), 227–241. 10.1002/wsbm.1331 PMC8363188 26990581

[B26] Mosmann T. (1983). Rapid colorimetric assay for cellular growth and survival: application to proliferation and cytotoxicity assays. J. Immunological Methods 65, 55–63. 10.1016/0022-1759(83)90303-4 6606682

[B27] Nakamura S. Watanabe T. Tanigawa T. Shimada S. Nadatani Y. Miyazaki T. (2018). Isoliquiritigenin ameliorates indomethacin-induced small intestinal damage by inhibiting NOD-like receptor family, pyrin domain-containing 3 inflammasome ActivationAnti-inflammatory effect of yam glycoprotein on lipopolysaccharide-induced acute lung injury via the NLRP3 and NF-κB/TLR4 signaling pathway. Pharmacology. 101 (5-6), 236–245. 10.1159/000486599 29393276

[B51] Niu X. Zang L. Li W. Xiao X. Yu J. Yao Q. (2020). Anti-inflammatory effect of Yam Glycoprotein on lipopolysaccharide-induced acute lung injury via the NLRP3 and NF-κB/TLR4 signaling pathway. Int. Immunopharmacol. 81, 106024. 10.1016/j.intimp.2019.106024 31784404

[B28] POWO (2021). Plants of the world online Availale at: http://powo.science.kew.org

[B29] Pu H. B. (2017). Clinical observation of Shenling Baizhu Powder in the treatment of infantile autumn diarrhea. Asia-Pacific traditional Med. 13 (11), 114–115. 10.11954/ytctyy.201711051

[B30] Qi W. (2019). Clinical study of Shenling Baizhu granule combined with western medicine in the treatment of rotavirus enteritis in children. New traditional Chin. Med. 51 (4), 94–96. 10.13457/j.cnki.jncm.2019.04.030

[B31] Reed L. J. Muench H. (1938). A simple method of estimating fifty per cent Endpoints12. Am. J. Hyg. 27 (3), 493–497. 10.1093/oxfordjournals.aje.a118408

[B32] Rosales-Martinez D. Gutierrez-Xicotencatl L. Badillo-Godinez O. Lopez-Guerrero D. Santana-Calderon A. Cortez-Gomez R. (2016). Rotavirus activates dendritic cells derived from umbilical cord blood monocytes. Microb. Pathogenesis 99, 162–172. 10.1016/j.micpath.2016.08.020 27554279

[B33] Ru J. Li P. Wang J. Zhou W. Li B. Huang C. (2014). TCMSP: a database of systems pharmacology for drug discovery from herbal medicines. J. Cheminform 6 (1), 13–18. 10.1186/1758-2946-6-13 24735618 PMC4001360

[B34] Saikh K. U. Morazzani E. M. Piper A. E. Bakken R. R. Glass P. J. (2020). A small molecule inhibitor of MyD88 exhibits broad spectrum antiviral activity by up regulation of type I interferon. Antiviral Res. 181, 104854. 10.1016/j.antiviral.2020.104854 32621945

[B35] Sen A. Sharma A. Greenberg H. B. (2017). Rotavirus degrades multiple interferon (IFN) type receptors to inhibit IFN signaling and protects against mortality from endotoxin in suckling mice. J. Virol. 92 (1), e01394–17. 10.1128/JVI.01394-17 29070687 PMC5730789

[B36] Sestak K. (2018). Non-human primate models of enteric viral infections. Viruses 10, 544–550. 10.3390/v10100544 PMC6213648 30301125

[B37] Shen J. Chen J.-J. Zhang B.-M. Zhao J. Chen L. Ye Q.-Y. (2019). Baicalin is curative against rotavirus damp heat diarrhea by tuning colonic mucosal barrier and lung immune function. Dig. Dis. Sci. 65 (8), 2234–2245. 10.1007/s10620-019-05977-w 31802384

[B38] Sherry B. (2009). Rotavirus and reovirus modulation of the interferon response. J. Interferon Cytokine Res. 29 (9), 559–567. 10.1089/jir.2009.0072 19694545 PMC2956631

[B39] Soderholm A. T. Pedicord V. A. (2019). Intestinal epithelial cells: at the interface of the microbiota and mucosal immunity. Immunology 158 (4), 267–280. 10.1111/imm.13117 31509239 PMC6856932

[B40] Szklarczyk D. Gable A. L. Lyon D. Junge A. Wyder S. Huerta-Cepas J. (2019). STRING v11: protein-protein association networks with increased coverage, supporting functional discovery in genome-wide experimental datasets. Nucleic Acids Res. 47 (D1), D607–D613. 10.1093/nar/gky1131 30476243 PMC6323986

[B41] Tate J. E. Burton A. H. Boschi-Pinto C. Parashar U. D. (2016). Global, regional, and national estimates of rotavirus mortality in children. Clin. Infect. Dis. 62 (Suppl. 2), S96–S105. 10.1093/cid/civ1013 27059362 PMC11979873

[B42] Troeger C. Khalil I. A. Rao P. C. Cao S. Blacker B. F. Ahmed T. (2018). Rotavirus vaccination and the global burden of rotavirus diarrhea among children younger than 5 years. JAMA Pediatr. 172 (10), 958–965. 10.1001/jamapediatrics.2018.1960 30105384 PMC6233802

[B43] Uchiyama R. Chassaing B. Zhang B. Gewirtz A. T. (2014). MyD88-mediated TLR signaling protects against acute rotavirus infection while inflammasome cytokines direct Ab response. Innate Immun. 21 (4), 416–428. 10.1177/1753425914547435 25213347 PMC7534410

[B44] Xu H.-Y. Zhang Y.-Q. Liu Z.-M. Chen T. Lv C.-Y. Tang S.-H. (2018). ETCM: an encyclopaedia of traditional Chinese medicine. *Nucleic Acids Res*. Oct. 47, D976–D982. 10.1093/nar/gky987 PMC6323948 30365030

[B45] Yang H. Oh K.-H. Kim H. J. Cho Y. H. Yoo Y. C. (2018a). Ginsenoside-Rb2 and 20(S)-Ginsenoside-Rg3 from Korean red ginseng prevent rotavirus infection in newborn mice. J. Microbiol. Biotechnol. 28 (3), 391–396. 10.4014/jmb.1801.01006 29316736

[B46] Yang J. Li S. Wang L. Du F. Zhou X. Song Q. (2018b). Ginsenoside Rg3 attenuates lipopolysaccharide-induced acute lung injury via MerTK-dependent activation of the PI3K/AKT/mTOR pathway. Front. Pharmacol. 9, 850–863. 10.3389/fphar.2018.00850 30116194 PMC6082957

[B47] Ye L. Jiang Y. Yang G. Yang W. Hu J. Cui Y. (2017). Murine bone marrow-derived DCs activated by porcine rotavirus stimulate the Th1 subtype response *in vitro* . Microb. Pathogenesis 110, 325–334. 10.1016/j.micpath.2017.07.015 28710013

[B48] Ye Y. Pei L. Ding J. Wu C. Sun C. Liu S. (2019). Effects of Platycodin D on S100A8/A9-induced inflammatory response in murine mammary carcinoma 4T1 cellsPatchouli alcohol activates PXR and suppresses the NF-κB-mediated intestinal inflammatory. Int. ImmunopharmacologyJournal Ethnopharmacology 67, 239–247. 10.1016/j.intimp.2018.12.008 30562685

[B49] Zhang H.-H. Yu W.-Y. Li L. Wu F. Chen Q. Yang Y. (2018). Protective effects of diketopiperazines from Moslae Herba against influenza A virus-induced pulmonary inflammation via inhibition of viral replication and platelets aggregation. J. Ethnopharmacology 215, 156–166. 10.1016/j.jep.2018.01.005 29309861

[B50] Zhou R. X. Song L. J. Shi X. Y. Wang X. T. Tan W. P. Lu H. Q. (2019). Study on the anti-rotavirus effect of Atractylodes I, II and III *in vivo* and *in vitro* . Chin. herbal Med. 50 (1), 104–110. 10.7501/j.issn.0253-2670.2019.01.017

