# Supplementary material for: Shenling Baizhu Powder Inhibits RV-SA11-Induced Inflammation and Rotavirus Enteritis via TLR4/MyD88/NF-κB Signaling Pathway
Source: Front Pharmacol. 2021 Apr 9;12:642685. doi: 10.3389/fphar.2021.642685 (PMC8062900; doi:10.3389/fphar.2021.642685)
Supplement: Supplementary file 1 [file datasheet1.pdf]

Supplementary Material

Table 1 Determination of virulence of RV-SA11 strain (n=3)

Concentration of virus	0	10^{-10}	10^{-9}	10^{-8}	10^{-7}	10^{-6}	10^{-5}	10^{-4}	10^{-3}	10^{-2}	10^{-1}
Average absorbance	1.37	1.22	1.18	1.32	1.13	1.05	0.82	0.72	0.61	0.57	0.45
Cell viability	1	0.81	0.78	0.88	0.75	0.70	0.55	0.48	0.41	0.38	0.30
Cytopathic rate	0	0.19	0.22	0.12	0.25	0.30	0.45	0.52	0.59	0.62	0.70

Table 2 The chemical constituents of SBP-containing serum identified by UPLC-Q-Orbitrap HRMS

Num-ber	Name	Formula	Molecular Weight	RT (min)
1	N1-(1,3,5-Trimethyl-1H-pyrazol-4-yl)-2-cyano-3-(dimethylamino)acrylamide	C12 H17 N5 O	247.14212	1.318
2	Isoliquiritigenin	C15 H12 O4	256.07323	12.036
3	2-Methoxyestrone	C19 H24 O3	300.17024	17.915
4	6-O-Pentopyranosyl-1-O-[(2,6,6-trimethyl-1-cyclohexen-1-yl)carbonyl]-β-	C21 H34 O11	526.22263	18.218
5	Oleanolic acid	C30 H48 O3	438.34975	19.468
6	Monoolein	C21 H40 O4	356.29247	20.026
7	18-β-Glycyrrhetinic acid	C30 H46 O4	470.3394	21.032
8	9(Z),11(E)-Conjugated Linoleic Acid	C18 H32 O2	280.24	21.869
9	Oleic acid	C18 H34 O2	282.25585	23.275

Table 3 The effects of drug - containing serum on the Cell viability of Caco-2 cells (%) (n=3)

drug-containing serum%	SBP	ribavirin
50	65.29±9.51	63.25±12.43
40	73.85±4.38	72.48±5.28
30	80.56±11.56	79.21±9.84
20	90.13±9.34	90.43±9.11
10	102.8±7.85	103.32±6.58
5	99.74±7.89	97.96±10.13
2.5	96.36±6.22	96.3±5.25

Table 4 The inhibitory effects of drug - containing serum on the RV SA11 in Caco-2 cells (%) (n=3)

drug-containing serum%	SBP	ribavirin
40	68.52±3.06	82.36±7.85
30	80.82±5.67	91.53±4.31
20	73.3±3.32	78.39±7.86
10	63.85±3.62	72.04±4.97
5	48.34±4.56	57.54±6.60
2.5	34.37±2.74	39.52±6.82

Table 5 The protein expressions of TLR4, MyD88 and NF - κ B p65 (n=3)

Group	TLR4	MyD88	NF - κ B p65
NC	0.0275±0.003	0.019±0.005	0.088±0.006
RV	0.738±0.041$^{\#\#}$	0.652±0.024$^{\#\#}$	0.655±0.038$^{\#\#}$
ribavirin	0.405±0.030**	0.421±0.034**	0.296±0.012**
SBP	0.452±0.013**	0.480±0.020**	0.261±0.016**

$^{\#\#}$ $P < 0.01$ vs. NC group; $^{*}P < 0.05$, ** $P < 0.01$ vs. RV group.

Table 6 The mRNA expressions of TLR4, MyD88, and NF-κB p65 (n=3)

Group	TLR4	MyD88	NF-κB P65
NC	1	1	1
RV	3.678±0.665$^{\#\#}$	4.015±0.25$^{\#\#}$	2.534±0.096$^{\#\#}$
ribavirin	1.785±0.191**	1.816±0.131**	1.556±0.076**
SBP	2.054±0.135**	1.510±0.144**	1.438±0.108**

$^{\#\#}$ $P < 0.01$ vs. NC group; $^{*}P < 0.05$, ** $P < 0.01$ vs. RV group.

Table 7 The expression of cytokines (pg/ml) (n=3)

Group	IL-Iβ	IL-6	TNF-α	IFN-β
NC	125.07±8.42	37.71±3.90	250.09±13.36	26.08±3.64
RV	228.43±14.70$^{\#\#}$	83.88±7.91$^{\#\#}$	446.17±12.22$^{\#\#}$	43.87±3.73$^{\#\#}$
ribavirin	166.06±8.32**	67.35±7.18*	292.34±11.91**	52.74±4.60*
SBP	172.89±8.59**	53.47±9.15**	308.38±16.63**	61.61±3.11**

$^{\#\#}$ $P < 0.01$ vs. NC group; $^{*}P < 0.05$, ** $P < 0.01$ vs. RV group.
